# Effect of wet-heparinized suction on the quality of mediastinal solid tumor specimens obtained by endoscopic ultrasound-guided fine-needle aspiration: a retrospective study from a single center

**DOI:** 10.1186/s12876-023-02845-w

**Published:** 2023-06-14

**Authors:** Bo Xu, Qian Lu, Rong Fang, Xiaojuan Dai, Haiyan Xu, Xiangwu Ding, Huawei Gui

**Affiliations:** 1 Department of Gastroenterology, Wuhan Fourth Hospital, Wuhan, China; 2 Department of Stomatology, Wuhan Children’s Hospital (Wuhan Maternal and Child Healthcare Hospital), Tongji Medical College, Huazhong University of Science and Technology, Wuhan, China; 3 Department of Pathology, Wuhan Fourth Hospital, Wuhan, China

**Keywords:** Mediastinal mass, EUS-FNA, Wet-heparinized suction, Specimen quality, Safety

## Abstract

**Background:**

Mediastinal lesions are diagnosed sometimes by endoscopic ultrasound-guided fine-needle aspiration (EUS-FNA). Wet-heparinized suction technique has been used to improve the quality of abdominal solid tumor samples obtained by EUS-FNA. The aim of the study is to assess the effect of wet-heparinized suction on the quality of mediastinal solid tumor samples and to evaluate the safety of the method.

**Methods:**

The medical records, EUS-FNA records, pathologic data, and follow-up data between the patients who suspected mediastinal lesions with wet-heparinized suction and conventional suction were retrospectively and comparatively analyzed. Adverse events at 48 h and 1 week after EUS-FNA were evaluated.

**Results:**

Wet-heparinized suction contributed to more tissue specimens (*P* < 0.05), superior tissue integrity (*P* < 0.05), and a longer length of white tissue core (*P* < 0.05). In addition, the more complete the tissue bar was, the higher the rate of successful sample (*P* < 0.05). Moreover, the total length of the white tissue bar at the first puncture was remarkably longer in the Experimental group (*P* < 0.05). No significant difference in red blood cell contamination in paraffin sections was found between the two groups (*P* > 0.05). There was no complication after discharge in both groups.

**Conclusion:**

Wet-heparinized suction can improve the quality of mediastinal lesion samples obtained by EUS-FNA and increase the success rate of sampling. In addition, it will not aggravate blood contamination in paraffin sections while ensuring a safe puncture.

**Supplementary Information:**

The online version contains supplementary material available at 10.1186/s12876-023-02845-w.

## Background

Mediastinal solid masses include mainly primary lesions, metastases, and benign/malignant lymph nodes, which can be diagnosed by some non-surgical methods such as percutaneous transthoracic core needle biopsy and endobronchial ultrasound-guided transbronchial needle aspiration. Endoscopic ultrasound-guided fine needle aspiration (EUS-FNA) is also an effective diagnostic approach [1] that has clear advantages when applied in diagnosis of mediastinal lymph nodes in lymph node stations 8 and 9 [2]. However, the conventional EUS-FNA can lead to poor specimen quality because of incomplete tissue structure, blood coagulation and other deficiencies [3]. At present, multiple techniques have been developed to improve the quality of puncture specimens, such as negative micro-pressure, fanning puncture and wet tap [4], but the effect is unsatisfactory. Previous studies pointed out that wet-heparinized suction for biopsy of a peritoneal lesion could increase the sample volume and reduce blood contamination [5–8]. Due to the distinct difference between the histological characteristics of peritoneal and mediastinal masses [9], this technique is poorly investigated in studies on mediastinal masses, especially on mediastinal lymph node. As reported, identification of the nature of mediastinal lymph node is essential for relevant tumor staging and treatment [10]. In this study, we aimed to assess the effect of the wet-heparinized suction technique in EUS-FNA for a mediastinal solid mass in aspects of tissue integrity, tissue yields and blood contamination.

## Methods

### Subjects

Medical records of 71 patients who were scheduled for an EUS-FNA for a mediastinal solid mass in Wuhan Fourth Hospital between August 2019 and April 2021 were harvested. Age, gender, EUS-FNA records, pathologic data (gross, histological and cytological diagnostic data), and follow-up data of patients included in this study were collected.

Patients were included if they were (1) aged > 18 years old, (2) suspected to have a mediastinal solid mass based on imaging findings (computed tomography(CT), magnetic resonance imaging(MRI), abdominal ultrasound) and had not undergone FNA. (3)Patients without coagulopathy (coagulopathy: international normalized ratio (INR) > 1.5 or blood platelet count < 8 × 104/mm3).(4)Patients who used antithrombotic drugs should have been stopped it for more than one week before FNA. Patients who had incomplete data were excluded from the study.

**Fig. 1:**
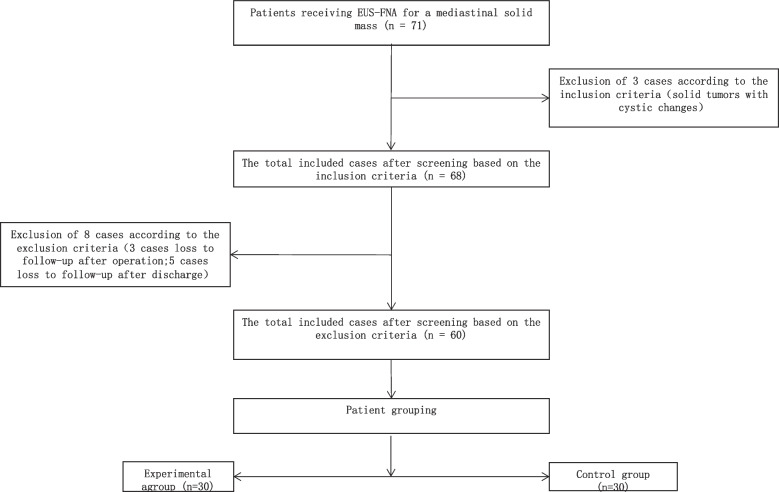
Patient screening workflow

Patients undergoing EUS-FNA all received the wet-heparinized suction technique after May 2020. Before that, we used the conventional suction technique (dry suction technique). Patients undergoing wet-heparinized suction technique were assigned to the Experimental group, while patients receiving conventional suction technique were classified into the Control group. All patients were fully informed of the puncture technique they would receive and the potential complications, and they all provided written informed consent before the procedure. Eventually, 60 patients were included in this study, including 30 in the Experimental group and the other 30 in the Control group (Fig. 1).

### Equipment and operator

EG-3870UTK echoendoscope; 19GSonoTipProContr puncture needle (GUS-33–21-019, Germany, Medi-Globe GmbH) [11].

The operator was a high-level endoscopist who had performed over 500 EUS-guided punctures.

#### Puncture techniques

(1) Conventional puncture: Tail of the empty puncture needle was connected to a pre-vacuum syringe. Three punctures were performed with a negative pressure (10 ml) applied by the syringe for 40–50 stabs each.

(2) Wet-heparinized puncture: The empty puncture needle was pre-filled with 100 U/ml heparinized saline and connected to a pre-vacuum syringe (containing 5 ml 100 U/ml heparin solution) via its tail. Three punctures were performed the same to the conventional method, and heparinized saline filling was provided prior to each puncture.

### Specimen processing

#### Collection

(1) The stylet was extracted, and the bar tissue specimen was pushed into a transparent flat dish (10 cm in diameter) containing 10% neutral buffered formalin fixative solution. Specimen quality was assessed by naked eyes with gentle, intermittent shaking of the dish. (vidoe1) (2) Air was drawn into a normal syringe (10 ml) to push the residual bloody tissue within the needle to a slide to prepare cell smears (3–6 smears each time). (3) The saline rinse solutions of the bloody tissue within the pre-vacuum syringe and of the puncture needle were collected.

### Measurement

**Fig. 2:**
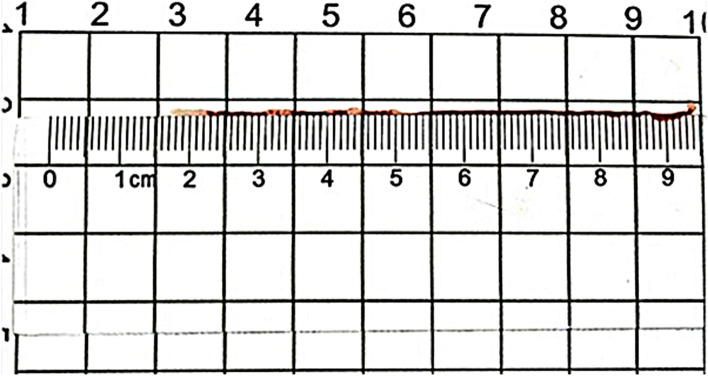
Measurement of white tissue

The puncture tissue bar consisted of a red part and a white part as visualized by the naked eyes [12]. Two parameters were measured by a ruler (Fig. 2): (1) the total length of the puncture tissue bar (the length required for pushing out the complete tissue bar by the stylet); (2) the length of consecutive tissue bar and the total length of the white part (white tissue core) at the first, second and third puncture.

### Pathologic examination

The tissue bar was paraffin-embedded and then processed for hematoxylin & eosin (HE) and immunohistochemical (IHC) staining. Cell smears were air-dried and Pap-stained. The rinse solutions of the bloody tissue within the pre-vacuum syringe and of the puncture needle were collected for liquid-based cytology (membrane-based and sedimentation).

### Result interpretation

- (1) Pathologic result interpretation: positive was interpreted by presence of benign/malignant tumor or heterotypic cells, while negative was interpreted by absence of benign/malignant tumor or heterotypic cells.
- (2) Standard of successful sampling: successful sampling was defined by conformity with histologic diagnosis, otherwise (i.e., insufficient sample volume, non-target tissue, necrosis, etc.), sampling failure was defined. A pathologist performed the assessment.
- (3) Tissue integrity: a consecutive tissue bar with white tissue cores, and the total length of the white tissue cores should be more than 10 mm.
- (4) Bloodiness of the paraffin Specimen processing Section: A 3-point scale was applied: 3, absence of red blood cells (RBC) /monolayer of RBC; 2, aggregates of RBC present < 1 high-powered field (HPF); 1, aggregates of RBC present > 1 HPF.

Two senior pathologists performed the result interpretation.

### Follow-up

Indicators (e.g., blood routine and chest CT findings), clinical symptoms and signs at 48 h following puncture were monitored, and complications (hemorrhage, fever, chest pain, and gastrointestinal perforation) were observed. The patients were further followed up for 1 week by in-hospital monitoring or out-hospital telephone interviews.

### Statistical analysis

SPSS 18.0.0 (IBM.cor) was used for statistical analysis. Shapiro–Wilk was applied to test the normality of quantitative data. In the context of normal distribution, data were expressed by mean ± SD and compared between two groups using an independent-sample t test; otherwise, data were shown as M (P25, P75) and compared via a Wilcoxon rank sum test. Qualitative data were displayed as case number and analyzed by a paired χ2 test. Fisher’s exact test was applied instead if the number was less than 5. Two-tailed *P* < 0.05 implied statistical significance.

## Results

### Patient general data

**Fig. 3:**
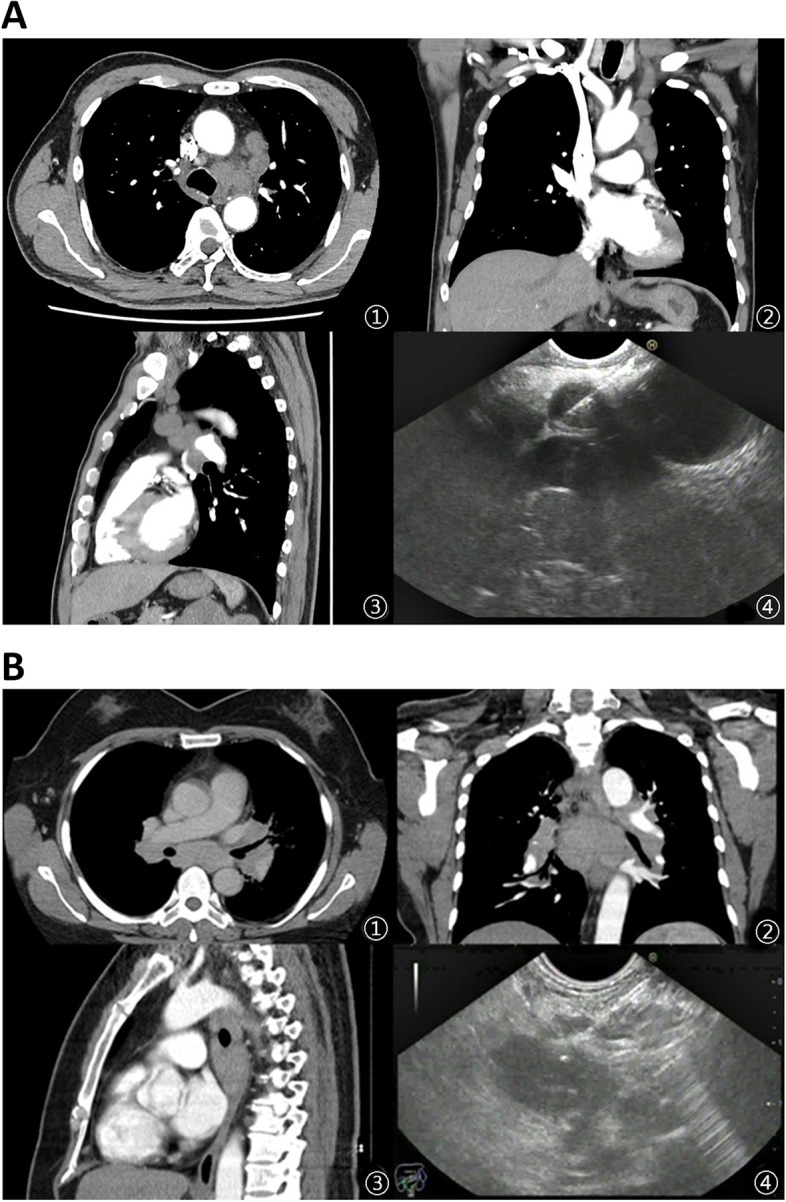
**a** A 56-year-old male patient. ①②③ Subcarinal lymph nodes are shown on CT scan. ④ Multiple round shaped lymph nodes were found in station 5 by EUS and EUS-FNA was performed. CT: computed tomography; EUS-FNA: endoscopic ultrasound-guided fine needle aspiration. **b** A 42-year-old female patient. ①②③ Left paratracheal lymph nodes are found on CT scan. ④ Oval hypoechoic lymph node was seen in station 4L by EUS and EUS-FNA was performed. CT: computed tomography; EUS-FNA: endoscopic ultrasound-guided fine needle aspiration

There were 16 males and 14 females in the Experimental group while 17 males and 13 females in the Control group (*P* > 0.05). The maximum diameter of the mass section had no statistical difference between the two groups (*P* > 0.05). Table 1 Lung squamous cell carcinoma was diagnosed in the majority of the patients. The mean length of the puncture tissue bars was 127 mm (Fig. 3).

**Table 1:** General data of patients

	**Experimental group**	**Control group**	***P***
Gender			> 0.05(.795)
Male	16	17	
Female	14	13	
Mean age (year)	59.63 ± 11.76	60.03 ± 11.23	> 0.05(.893)
Maximum diameter of mass section (mm)	16.60 ± 4.06	17.43 ± 3.58	> 0.05(.403)
antithrombotic treatment, n(%)	8(22.66)	10(33.33)	> 0.05(.573)
**Diagnosis**			> 0.05(.621)
Lung squamouscell carcinoma	22	19	
Metastasis of non-lung malignancy	1	2	
Lymphoma	2	0	
Tuberculosis	2	3	
Sarcoidosis	0	1	
Benign nodule	3	5	
**Tumor site**			> 0.05(.776)
Trachea carina (station 4L, station 5)	24	26	
Main pulmonary artery window (station 7)	4	3	
Para-esophageal (station 9)	2	1

### Comparison of puncture outcomes

In comparison to the Control group, the Experimental group obtained more successful tissue samples, and the more complete a puncture tissue par was, the more likely the sampling was successful (*P* < 0.05) (Table 2). We found that the Experimental group was superior to the Control group with respect to the completeness of the tissue bar punctured (*P* < 0.05) (Table 2).

**Table 2:** Comparison of specimen adequacy and tissue completeness between groups

	**Experimental group**	**Control group**	***P***
Number of punctures, n	90	90	
Successful specimen, n (%)	53(58.9)	38(42.2)	< 0.05 (.025)
Completeness of the tissue, n (%)	49(54.4)	30(33.3)	< 0.05(.004)

In addition, the total length of the tissue bar (*P* < 0.05) and the white tissue core (*P* < 0.05) in the Experimental group were significantly longer compared with those in the Control group. The total length of the white tissue core at the first puncture was remarkably longer in the Experimental group (-, *P* < 0.05), whereas those at the second (-*P* > 0.05) and third (*P* > 0.05) punctures had minor differences between the two groups (Table 3).

**Table 3:** Comparison of puncture tissue bar length and RBC contamination

	**Experimental group**	**Control group**	***P***
Total length of tissue bar (mm) (mean ± SD)	398.70 ± 43.59	375.26 ± 54.12	< 0.05(.000)
Total length of white tissue core (mm) (mean ± SD)	59.93 ± 11.04	45.23 ± 13.75	< 0.05(.000)
Total length of white tissue core at the first puncture (mm) (mean ± SD)	32.73 ± 9.43	16.10 ± 4.63	< 0.05(.000)
Total length of white tissue core at the second puncture (mm) (mean ± SD)	15.87 ± 4.50	18.17 ± 6.81	> 0.05(.129)
Total length of white tissue core at the third puncture (mm) (mean ± SD)	11.33 ± 3.18	10.97 ± 4.16	> 0.05(.702)
RBC contamination (point) (mean ± SD)	2.086 ± 0.15	2.032 ± 0.12	> 0.05(.136)
RBC contamination at the first puncture (point) (mean ± SD)	2.290 ± 0.12	1.533 ± 0.18	< 0.05(.009)
RBC contamination at the second puncture (point) (mean ± SD)	2.060 ± 0.21	2.203 ± 0.24	> 0.05(.407)
RBC contamination at the third puncture (point) (mean ± SD)	1.630 ± 0.24	2.363 ± 0.24	> 0.05(.860)

No statistical difference with respect to the RBC contamination in paraffin sections was noted between the two groups (*P* > 0.05) (Table 3).

### Complications and follow-up

After surgery, a total of 5 patients presented with chest pain and recovered within 1 week after medical conservative treatment. There was no complication 1 week after discharge in both groups. No difference in the incidence of complication was found between the two groups (*P* > 0.05) (Table 4).

**Table 4:** Comparison of complications between groups (n)

Chest pain	Experimental group	Control group	*P*
			> 0.05 (.643)
Yes	3	2	
No	27	28	

## Discussion

EUS-FNA with the wet suction method is capable of increasing the sample volume [13–15], as a water film can be formed in the wet tube wall to avoid adhesion and thereby to increase the sample volume. Heparin can be also employed in EUS-FNA to increase the sample volume [5, 6, 13, 16] by preventing the blood coagulation-induced adhesion between tissue and needle tube wall. At present, few heparin-based studies have included mediastinal solid masses, particularly with respect to the effect of heparin on the histomorphological features of the specimen obtained by and the rate of successful sampling via puncture.

**Fig. 4:**
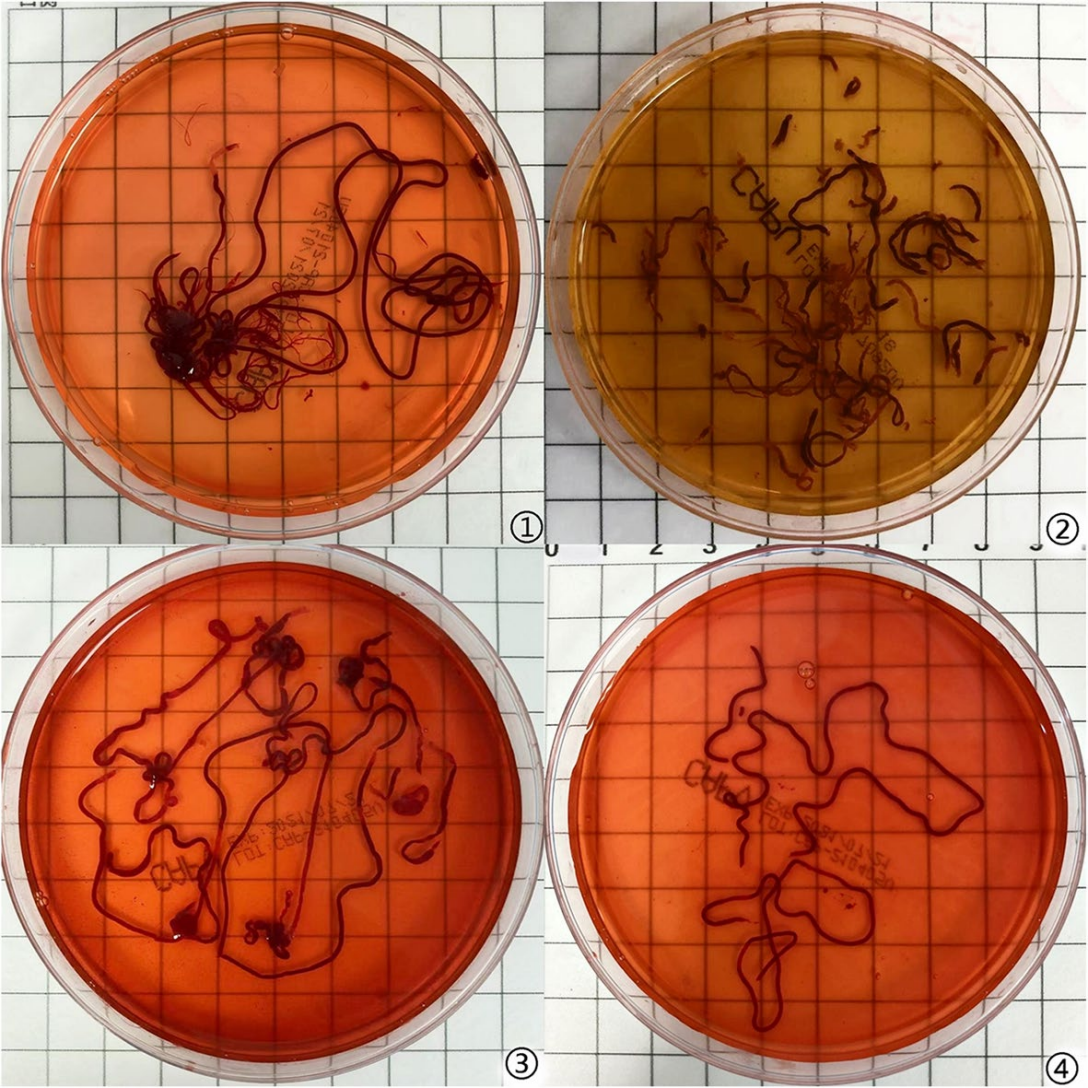
①②: The first and third punctures in the Control group.③④: The first and third punctures in the Experimental group

Pathologic diagnosis is made mainly based on positive cell identification and histomorphometric findings, while the latter is more valuable. A complete tissue bar obtained by FNA is particularly important for some lesions requiring identification in histomorphology [17, 18]. In the present study, we found that wet-heparinized suction contributed to more complete tissue bars (Fig. 4), suggesting that heparin can stabilize the structure of tissue bar to prevent breakage. Additionally, a higher rate of successful sampling was noticed in the Experimental group (*P* < 0.05), which implied a higher success rate upon complete tissue sampling. Moreover, the sample volume in the Experimental group was larger, consistent with the studies of Mok SRS et al. [5, 13]. This finding suggests that there are some common features of experience with EUS-FNA performed in different sites. It is generally believed that a larger sample volume performs better in final diagnosis. Bilaçeroğlu S et al. [19] believed that sufficient sample volume is an important factor that facilitates diagnosis, since it can not only fulfill the criteria for a pathologic diagnosis but also be used for adjuvant examinations such as IHC staining [20, 21]. Here, we selected a 19G needle for puncture, as a large-bore needle contributes to a higher positive rate and accuracy in diagnosis of mediastinal lesions and more ideal tissue specimens [22–25]. The results above indicate that use of heparin in puncture can improve the specimen quality from two aspects: tissue completeness and volume.

This study also demonstrated that the specimens obtained with the method of wet-heparinized suction consisted of mainly a red part and a white part. Generally, the red part is composed of blood clots or mixed tissue, while the white part comprises commonly target tissue [26, 27]. Therefore, the white part (white tissue core) is more significant in diagnosis of mediastinal lesions. During our experiment, the specimens in the dish were shaken to accelerate fixation, and the uncoagulated blood was found as dissolved in the fixative solution. Statistical analysis revealed that the total length of white tissue core in the Experimental group was remarkably longer than that in the Control group. Combining the findings, we speculate that heparin application prevents blood coagulation in tissue, while the uncoagulated blood is more soluble in the fixative solution. Factors such as crushed tissues and hemorrhage within the target lesion can also affect the quality of the specimen obtained via puncture [28]. In this context, we reasoned that the anticoagulant effect of heparin may be reduced as more punctures are applied. Our study found that there were no significant differences in the length of white tissue at the second and third punctures between the two groups (*P* < 0.05), which confirmed our speculation. Therefore, we suggested that wet-heparinized suction technique should be applied for the first needle puncture, which could improve the quality of the specimen and not significantly increase the complexity of the operation.

Blood contamination in paraffin sections can significantly affect the result of pathological diagnosis, and current studies on the effect of heparin use in puncture are controversial [6, 29]. A recent meta-analysis demonstrated that heparin use had no significant effect on blood contamination in paraffin sections [8]. In this context, we found that there was a statistically significant difference in the blood contamination between the two groups in the first puncture, with less blood contamination in the Experimental group than in the Control group (*P* < 0.05). There was no statistically significant difference in the blood contamination between the two groups in the second and third punctures (*P* > 0.05). We speculated that the Control group used a dry empty puncture needle in the first puncture. During the second and third punctures, the needle lumen was flushed with saline, which moistened the needle lumen. Therefore, the Control group was performing a technique similar to the wet-suction technique after the second puncture. The present study quantitatively analyzed the blood contamination in paraffin sections and eventually found no remarkable difference between the two groups (*P* > 0.05).

During the short-term follow-up, no complications were noticed, in addition to chest pain in 5 patients that recovered after temporarily medical treatment. There were eighteen patients who had received antithrombotic treatment, with eight patients in the Experimental group and ten patients in the Control group. Five patients who eventually experienced chest pain did not receive antithrombotic treatment. Statistically, there was no significant difference between the two groups in terms of the incidence of complications (*P* > 0.05), which suggested that heparin is safe in mediastinal puncture without causing severe complications [5, 30, 31].

To sum up, application of heparin in puncture for a mediastinal lesion can increase the completeness and volume of the tissue samples and decrease blood coagulation within tissues without aggravating RBC contamination in sections, which is safe. High-quality specimens enabled a transition from initial cytological diagnosis to histological diagnosis and further development towards genetic diagnosis. For example, next-generation sequencing can be performed on endoscopic ultrasound-guided tissue acquisition specimens to guide clinical diagnosis. Next-generation sequencing also could be used to establish clinical models to guide treatment, and promote the development of precision medicine [32].

However, there are some deficiencies in the current study. First, the white tissue bar sampled via puncture might contain fibrotic tissues when there is severe fibrosis around the mass [12, 26, 33], which may affect the accuracy of the result. Besides, whether the presence of fibrosis in the tissue sampled indicates tumor remains unclear. Therefore, further research is warranted to investigate the effect of fibrosis on the result of puncture. Second, the long-term outcome of some patients with a benign mass remains unknown, and therefore the accuracy of puncture cannot be assessed. Finally, the sample size is small, requiring large-scale randomized controlled trials to further validate the results of the study.

## Conclusion

This study identified that wet-heparinized suction in EUS-FNA can improve the quality of mediastinal lesion samples and increase the success rate of sampling. In addition, it can reduce the intrinsic and extrinsic tissue coagulation and not aggravate blood contamination in paraffin sections while ensuring a safe puncture.

## Supplementary Information

**Additional file 1.**

## Data Availability

The datasets generated during and analyzed during the current study are obtained from the His system of the Wuhan Fourth hospital, and available from the corresponding author on reasonable request.

## Abbreviations

EUS-FNA: Endoscopic ultrasound-guided fine-needle aspiration
CT: Computed tomography
MRI: Magnetic resonance imaging
HE: Hematoxylin & eosin
ICH: Immunohistochemical
RBC: Red blood cells
HPF: High-powered field
